# Repair Effects of Astragalus Polysaccharides with Different Molecular Weights on Oxidatively Damaged HK-2 Cells

**DOI:** 10.1038/s41598-019-46264-y

**Published:** 2019-07-08

**Authors:** Jin Han, Da Guo, Xin-Yuan Sun, Jian-Min Wang, Jian-Ming Ouyang, Bao-Song Gui

**Affiliations:** 1 Department of Nephrology, the Second Hospital of Xi’an Jiaotong University, Xi’an, 710004 China; 2 Institute of Biomineralization and Lithiasis Research, Jinan University, Guangzhou, 510632 China

**Keywords:** Glycobiology, Urology

## Abstract

This study investigated the repair effects of three Astragalus polysaccharides (APSs) with different molecular weights (Mws) on injured human renal proximal tubular epithelial (HK-2) cells to reveal the effect of Mw of polysaccharide on cell repair. A damage model was established by injuring HK-2 cells with 2.6 mM oxalate, and APS0, APS1, and APS2 with Mw of 11.03, 4.72, and 2.61 KDa were used to repair the damaged cells. After repair by APSs, the morphology of damaged HK-2 cells gradually returned to normal, the destruction of intercellular junctions recovered, intracellular reactive oxygen species production amount decreased, and their mitochondrial membrane potential increased. In addition, the cell cycle progression gradually normalized, lysosome integrity increased, and cell apoptotic rates obviously declined in the repaired cells. All three APSs could promote the expression of Keap1, Nrf2, SOD1, and CAT. In addition, the expression levels of inflammation markers containing MCP-1 and IL-6 decreased after APS repair. We deduced that APSs exert their repair function by activating the Nrf2–Keap1 signaling pathway and inhibiting inflammation. Among the APSs, APS1 with a moderate Mw provided the strongest repair effect. APSs may have a preventive effect on kidney stones.

## Introduction

Radix astragali has been widely used as a diuretic for thousands of years and is widely distributed in temperate regions worldwide^1^. Astragalus polysaccharides (APSs) are the main medicinal active ingredients in *Astragalus membranaceus*. APSs exert antioxidant, antitumor, anti-aging, cardiovascular protective, liver protective, and kidney protective effects^2,3^. Yue *et al*.^4^ have shown that APSs can protect the mitochondrial membrane potential (ΔΨm) and lysosomes of PC12 cells and inhibit their apoptosis.

Many studies have shown that APSs are mainly composed of nine ratios of monosaccharides, namely, glucose (Glc), galactose (Gal), arabinose, rhamose (Rha), mannose, xylose (Xyl), fucose, fructose, and ribose, and contain glucuronic acid and galacturonic acid^5^. APSs prepared using different raw materials or purification processes differ in structure and conformation. For example, Li *et al*.^6^ used thin-layer chromatography (TLC) and Sephadex G-100 chromatography to isolate a polysaccharide with a Mw of 3.6 × 10^4^ Da from the roots of *A. membranaceus*. The chain consists of (1 → 6)-α-D-Glcp and α-D-(1 → 4)-Glc with branches attached to O-6. Fu *et al*.^7^ obtained an APS with a Mw of 3.01 × 10^5^ Da from Mongolian *A. membranaceus*. ^1^H NMR and ^13^C NMR spectra showed that the main chain is composed of 1,3-linked β-D-Gal residues and the branch chains have 1,5-linked β-Xyl, β-D-Gal, β-Glc, 1,4-linked β-Gal, 1,6-linked α-Gal, 1,2-linked α-Rha, and 1,2,4-linked α-Rha residues.

The cellular damage mediated by reactive oxygen species (ROS) is associated with the pathogenesis of many diseases, such as myocardial ischemia-reperfusion injury, atherosclerosis, and hypertension^8^. Studies have shown that high uric oxalate is the main component that damages renal epithelial cells. High concentrations of oxalate can promote excessive ROS production and cause renal epithelial cell damage by oxidizing lipids and other biological macromolecules^9^. In addition, nano/micron-sized calcium oxalate monohydrate (COM) and dihydrate (COD) can cause oxidative lesions to renal epithelial cells, leading to decreased mitochondrial membrane potential and increased ROS level^10^. Nano-sized crystals cause organelle injury faster than micron-sized crystals, and COM crystals show more obvious time-dependent effects than the same-sized COD crystals.

Polysaccharides reportedly exert protective and repair effects on oxidative damage to cells, which reduces the incidence of kidney stones^11^. In our previous study^12^, we investigated the antioxidant and repair abilities of green tea polysaccharide (TPS) with various Mws on injured renal epithelial cells. Tea is a common food ingredient and popular non-alcoholic beverage in Asia^13^. Four kinds of TPSs with various Mws showed antioxidant and repair abilities on oxidatively damaged renal cells. However, the detailed mechanism and the signaling pathway of the polysaccharide in repairing damaged renal epithelial cells remain unclear.

The bioactivity of polysaccharides is closely associated with their Mw^14–16^. Sun *et al*.^14^ prepared three polysaccharide fractions with different Mws coded as EPS-1, EPS-2, and EPS-3 by degrading *Porphyridium cruentum* polysaccharides with an original Mw of 2918.7 KDa (EPS-0). The Mws of the three degraded polysaccharides are 256.2, 60.66, and 6.55 KDa, respectively. EPS-0 exerts negative antioxidant activity, but all three degraded polysaccharide fractions possess remarkable inhibitory effect on oxidative damage. Among the three polysaccharide fractions, EPS-3 with the lowest Mw shows the strongest antioxidant activity and EPS-1 with the highest Mw exhibits the weakest activity. Ying *et al*.^15^ extracted three polysaccharide fractions from Liupu tea, namely LTPS-30 (7.1 KDa), LTPS-50 (6.9 KDa), and LTPS-70 (6.6 KDa). All three fractions can improve the cell viability of injured human umbilical vein endothelial cells (HUVECs). Moreover, LTPS-70 with the smallest Mw among the tested polysaccharide fractions exhibits the greatest antioxidant and repair effects on injured HUVECs. Trombetta *et al*.^16^ showed that *Opuntia ficus-indica (L.)* cladode polysaccharide promotes skin epithelial cell healing in injured mice when the Mw is more than 10^4^ Da, and the components with a Mw of 10^4^–10^6^ Da are more active than those components with a Mw of 10^6^ Da. Thus, polysaccharides with a certain Mw exert optimal bioactivity.

In the present study, three APSs with different Mws were used to repair oxidatively damaged renal tubular epithelial cells, the effects of polysaccharide Mw on cell repair were investigated, and changes in the protein expression, inflammatory factors, and signal pathways of HK-2 cells before and after repair were discussed.

## Experiments

### Reagents and apparatus

The original astragalus polysaccharide (APS0) was purchased from Beijing Puboxin Biotechnology Co., Ltd. The polysaccharide content was ≥95%. The degradation polysaccharides APS1 and APS2 and their structure were obtained according to previous study^10^. The Mws of APS0, APS1 and APS2 were 11.03 K, 4.72 K and 2.61 KDa, respectively (Table 1). Polysaccharide structure was characterized by ^1^H NMR, ^13^C NMR, FT-IR, and GC/MS. The main chain structure of APS did not obviously change before and after degradation. The three polysaccharides were composed of glucose, arabinose, rhamnose, and galactose.

**Table 1:** Molecular weight and -COOH group contents of APSs.

Sample	Molecular weight Mw/KDa	Content of -COOH/%
APS0	11.03	16.8
APS1	4.72	17.2
APS2	2.61	16.2

HK-2 cells were provided by Shanghai Cell Bank (Shanghai, China). Fetal bovine serum and DMEM culture medium (HyClone Biochemical Products Co. Ltd., Beijing, China). Cell proliferation assay kit (CCK-8, Dojindo Laboratory, Kumamoto, Japan). Reactive oxygen species (ROS) assay kit, mitochondrial membrane potential assay kit (JC-1) and Annexin V–FITC/propidium iodide assay kit (Beyotime Bio-Tech Co. Ltd., Shanghai, China). Mouse monoclonal anti-MCP-1, rabbit monoclonal anti-IL-6, rabbit monoclonal anti-Nrf2, rabbit monoclonal anti-ZO-1, mouse monoclonal anti-Keap1, rabbit monoclonal anti-SOD1 and rabbit monoclonal anti-CAT were purchased from Proteintech Group (Wuhan, China) and diluted 1:3000 in 5% skim milk/PBS.

The apparatus used include Enzyme Mark Instrument (SafireZ, Tecan, Switzerland), an upright electric fluorescence microscope (22DI-E-D282, Leica, Germany), flow cytometry (FACS Aria, BD company, USA).

### Cell culture and cytotoxicity detection of APSs

HK-2 cells were grown in DMEM-F12 medium supplemented with 10% fetal bovine serum at 37 °C and 5% CO_2_ moist environment. When 80–90% of the confluent monolayer is reached, cells are gently blown after trypsin digestion for the next cell experiment.

Cells were seeded in 96-well plate at a density of 1 × 10^5^ cells/mL and incubated for 24 h. Then, 200 μL of APS with different Mws of 0, 20, 60 and 100 μg/mL were supplemented. After the cells were incubated for 24 h, the OD value was measured at 450 nm using an enzyme labeler to calculate cell viability. Cell viability is calculated by the following formula:

$${\rm{Cell}}\,{\rm{viability}}( \% )={{\rm{OD}}}_{{\rm{treatment}}{\rm{group}}}/{{\rm{OD}}}_{{\rm{control}}{\rm{group}}}\times 100.$$

### Cell viability assay after APS repair of damaged HK-2 cells

200 microliters of cells suspension with a concentration of 1 × 10^5^ cells/mL was inoculated per well in 96-well microplates. The experiment is divided into four groups: (1) Control group of background: cell-free medium group; (2) Normal control group: normal cells were incubated in serum-free medium; (3) Damaged group: cells were damaged by 2.6 mM oxalate for 3.5 h; (4) Repair group: 20, 40, 60, 80, and 100 μg/mL APSs with different Mws were supplemented into damaged group for repairing for 10 h. The OD value was measured at 450 nm by an enzyme labeler to calculate cell viability.

### Cell morphology observation after APS repair of damaged HK-2 cells

Cells were seeded in 6-well plate at a density of 1 × 10^5^ cells/mL and incubated for 24 h. We set up three experimental groups: (1) Normal control group: normal cells were incubated in serum-free medium; (2) Damaged group: cells were damaged by 2.6 mM oxalate for 3.5 h; (3) Repair group: APS0, APS1 and APS2 with different Mws of 60 μg/mL were added to the damaged group for repair for 10 h. The cellular morphological changes were observed under microscope.

### Reactive oxygen species (ROS) detection

Following the cell incubations described as above, the cells were treated according to the ROS test kit instructions. Briefly, cells were stained with 500 μL of diluted DCFH-DA in serum-free medium and incubated for 30 min. After removal of excess dye, the ROS level was observed by a fluorescence microscope and the fluorescence intensity was detected by a microplate reader.

### Measurement of mitochondria membrane potetial (ΔΨm)

Following the cell incubations described as above, the ΔΨm of cells was detected according to our previous study^12^. Briefly, the treated cells were stained with 200 μL of JC-1 for 15 min in the dark, then the ΔΨm was detected by flow cytometry.

### Changes in lysosomal integrity before and after repair

According to the procedure in our previous study^12^, lysosomal integrity was detected by AO staining. Briefly, the cells were preloaded with AO (5 μg/mL) for 15 min, then the cells were treated following the cell incubations described as above. The AO distribution in cells was observed by fluorescence microscope, and the red and green flurescence were detected by microplate reader.

### Cell cycle assay

After synchronization, cells were incubated as above. According to the procedure in our previous study^12^, analysis of cell cycle was performed by flow cytometry after PI staining. Briefly, cells were fixed using 70% ethanol at 4 °C overnight, then the treated cells were resuspended in 200 μL of PI dye and kept for 15 min at 37 °C. The amount of PI-labeled DNA was analyzed by flow cytometry.

### Apoptosis detection before and after repair

Following the cell incubations described as above, the treated cells were stained by Annexin V-FITC (5 μL) for 10 min under dark environment. Then the treated cells were stained by PI (5 μL) after being resuspended in binding buffer. Finally, the apoptosis rates were analyzed by a flow cytometer.

### Western blot analysis

After repair for 10 h, the treated cells were washed for 2–3 times and lysed by adding an appropriate volume of RIPA lysate^17^. The cells and reagents were scraped off with a cell scraper, collected in a 1.5 mL centrifuge tube, and ice-bathed for 30 min, during which time the pipette was repeatedly tapped to ensure complete cell lysis. The cells were centrifuged at 12,000 rpm and 4 °C for 10 min, and then the supernatant was collected. Total protein (50 μg) was loaded onto a 12% tris-glycine SDS–polyacrylamide gel, which was then run at 120 V for 1 h before the protein was transferred to a polyvinylidene difluoride (PVDF) membrane. The membrane was blocked with 5% defatted milk for 2 h, and primary antibodies against MCP-1, IL-6, Keap1, Nrf2, SOD1, CAT, ZO-1, and GAPDH were incubated overnight at 4 °C and then washed five times using TBST buffer. The second antibody was diluted 3000-fold with TBST and incubated for 1 h at room temperature. After washing the membrane 5 times, 1 mL of ECL was added and incubated in the cassette for 5 min to scan using a CCD fluorescence microscope. Horizontal scanning densitometry was performed on Western blots using acquisition into Adobe PhotoShop (Adobe Systems Inc., San Jose, CA).

### Statistical analysis

Experimental data are expressed as mean ± standard deviation (x ± SD). Statistical analysis of the experimental results was performed using SPSS 13.0 software. The difference of means was analyzed using one-way ANOVA, followed by Tukey’s post hoc test. P < 0.05 was considered statistically significant; p < 0.01 was considered extremely significant.

## Results

### Toxicity assessment of APSs on normal HK-2 cells

**Figure 1:**
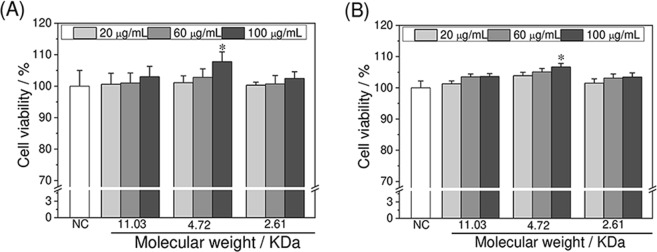
Cytotoxicity of different Mw APS on HK-2 cells detected by CCK-8 (**A**) and MTT (**B**) methods, respectively. HK-2 cells were incubated with polysaccharide for 24 h. Compared with normal control (NC) group, *p < 0.05.

The cytotoxicity of APSs (APS0, APS1, and APS2) with Mws of 11.03, 4.72, and 2.61 KDa on HK-2 cells was comparatively evaluated by the CCK-8 (Fig. 1A) and MTT (Fig. 1B) methods. For the CCK-8 method, cell viability was above 100% when HK-2 cells were treated with various concentrations of polysaccharides (20, 60, and 100 μg/mL) for 24 h, and all of them slowly increased (101.2–107.8%) as the concentrations of polysaccharides were increased. At concentrations below 100 μg/mL, the three polysaccharides with different Mws were non-toxic to HK-2 cells and exerted a promoting effect. MTT assay showed similar results (Fig. 1B) to CCK-8 assay. Table 2 lists the minor differences between the results obtained by the two methods.

**Table 2:** Comparison of CCK-8 assay and MTT assay to detect cytotoxicity of different Mw APS on HK-2 cells.

Groups	Concentration/μg/mL	Cell viability/%	
		CCK-8	MTT
NC		100 ± 5.0	100 ± 2.2
ASP0	20	100.6 ± 3.5	101.3 ± 0.9
	60	101 ± 3.2	103.5 ± 1.1
	100	103 ± 3.3	103.6 ± 1.4
ASP1	20	101.1 ± 2.2	104.9 ± 2.9
	60	102.8 ± 2.7	105.1 ± 0.6
	100	107.8 ± 3.1	106.7 ± 0.5
ASP2	20	100.3 ± 1	100.3 ± 0.3
	60	100.7 ± 2.7	101.5 ± 1.2
	100	102.5 ± 2.1	103.4 ± 1.1

### APS repair increased viability of damaged HK-2 cells

**Figure 2:**
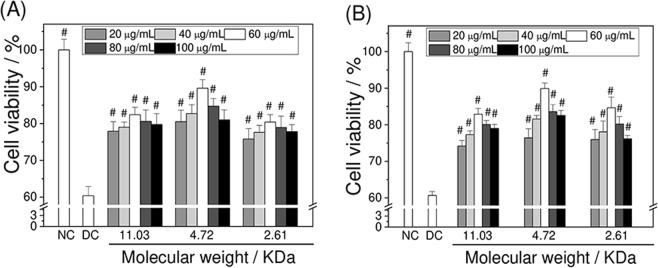
Changes in cell viability of damaged HK-2 cells after being repaired by different Mw APS detected by CCK-8 (**A**) and MTT (**B**) methods, respectively. Normal Control (NC); Oxalate damaged control (DC). Oxalate concentration: 2.6 mM. Damaged time: 3.5 h; Repaired time: 10 h. Compared with DC group, *p < 0.05, ^#^P < 0.01.

The cell viabilities of oxalate injured HK-2 cells after repair by various concentrations of APS0, APS1, and APS2 were comparatively detected by the CCK-8 (Fig. 2A) and MTT methods (Fig. 2B). The optimal repair effect of all polysaccharides was achieved at 60 μg/mL. For instance, the cell viability detected by CCK-8 method of the injured group increased from 60.4% to 80.5%, 82.8%, 89.6%, 84.7%, and 81.0% after repair with APS1 at 20, 40, 60, 80, and 100 μg/mL concentrations, respectively.

The Mw of APSs affected their repair effect. APS1 with a moderate Mw provided the best repair effect under the same concentration. For example, the cell viability of damaged group increased from 60.4% ± 4.5% to 82.4% ± 2.0%, 89.6% ± 2.8%, and 80.4% ± 2.0% after repair with APS0, APS1, and APS2, respectively, at 60 μg/mL concentration. The results of the MTT method (Fig. 2B) were similar to those of the CCK-8 method (Fig. 2A). Table 3 lists the changes in cell viabilities detected by the CCK-8 and MTT methods. The above results suggest that the repair capability of APSs correlates with their concentrations and Mw.

**Table 3:** Comparison of CCK-8 and MTT assays to detect the changes in damaged HK-2 cells activity after APS repair with different Mws.

Groups	Concentration/μg/mL	Cell viability/%	
		CCK-8	MTT
NC		100 ± 2.9	100 ± 2.4
DC		60.4 ± 2.5	60.7 ± 1.1
ASP0	20	77.9 ± 2.6	74.2 ± 1.5
	40	79 ± 1.4	77.3 ± 1.0
	60	82.4 ± 2.0	82.9 ± 1.6
	80	80.6 ± 3.1	80.1 ± 1.1
	100	79.7 ± 3.0	78.9 ± 1.1
ASP1	20	80.5 ± 3.1	76.4 ± 2.5
	40	82.7 ± 2.4	81.6 ± 0.9
	60	89.6 ± 2.3	89.9 ± 1.4
	80	84.7 ± 2.1	83.6 ± 1.9
	100	81 ± 2.7	82.5 ± 1.3
ASP2	20	75.8 ± 2.8	75.9 ± 2.8
	40	77.6 ± 1.9	78.1 ± 3.0
	60	80.4 ± 2.0	84.6 ± 2.9
	80	78.9 ± 3.1	80.2 ± 2.0
	100	77.8 ± 1.9	76.1 ± 0.9

### APS repair restored cell morphology

**Figure 3:**
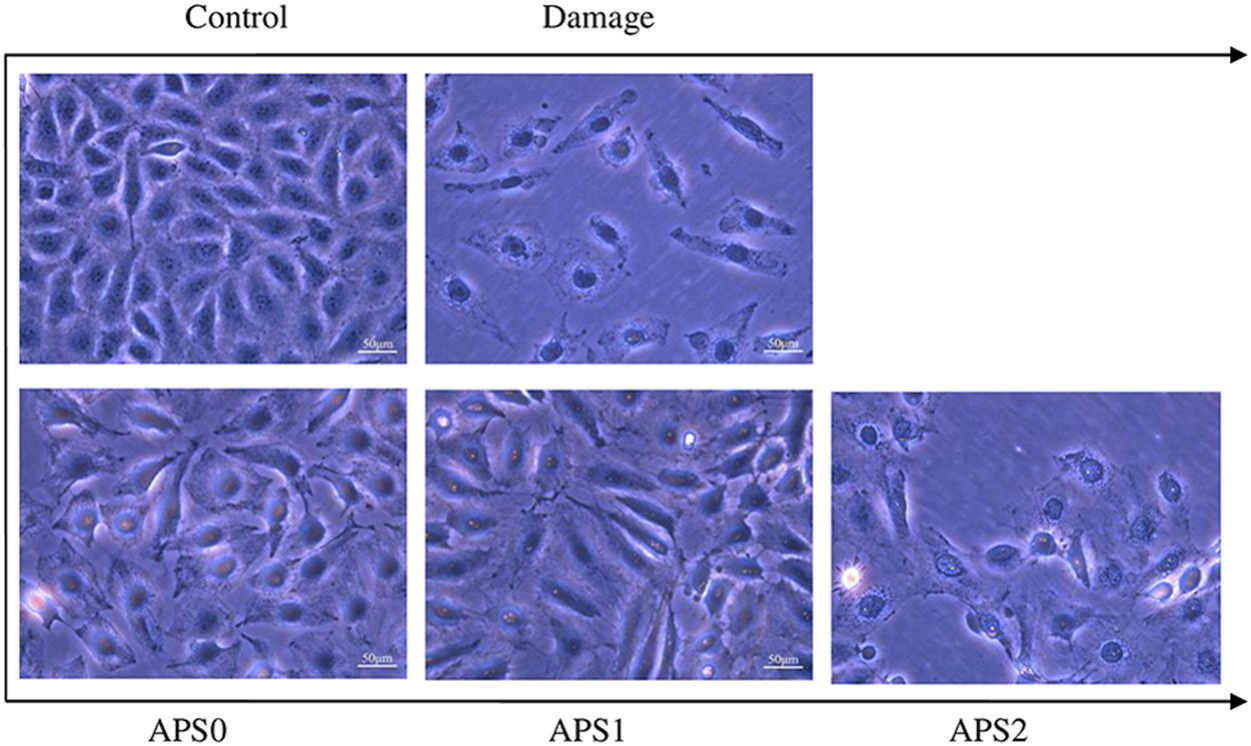
Changes of cell morphology in 2.6 mM oxalate damaged HK-2 cells after repair by different Mw APS (60 μg/mL) for 10 h.

The changes of cell morphology of oxalate damaged cells before and after APS repair were observed with a microscope (Fig. 3). The normal cells are tightly connected and the cells are plump. However, oxalate-injured cells lost their natural morphology and their volume decreased obviously. After repair by APS0, APS1 and APS2 at 60 μg/mL, cell number increased and their morphologies gradually returned to normal. After being repaired by APS1 with a moderate Mw, the morphology of repaired cells is close to normal.

### APS repair decreased intracellular ROS generation

Excessive ROS induced by harmful stimulation will cause oxidative damage to biomolecules (such as lipids, DNA, proteins, etc.), thus inducing cell injury or death^18^.

**Figure 4:**
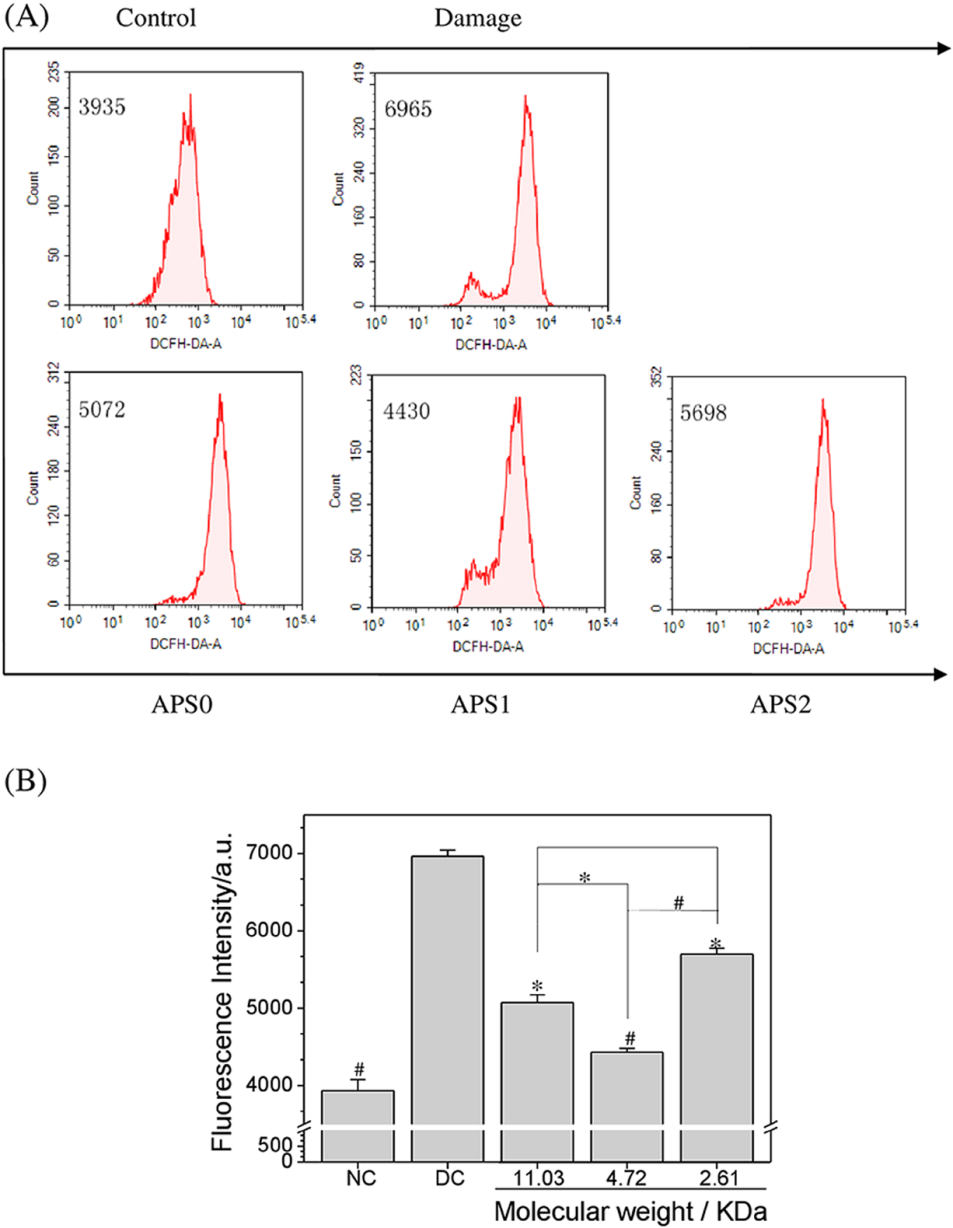
Changes in ROS level of 2.6 mM oxalate damaged HK-2 cells after repair by different Mw APS (60 μg/mL) for 10 h. (**A**) Histogram of intracellular ROS; (**B**) quantitative results of intracellular ROS. Compared with DC group, ^*^P < 0.05; ^#^P < 0.01.

The changes in intracellular ROS were detected by DCFH-DA staining (Fig. 4). Damaged cells exerted strong fluorescent intensity, which indicated that massive ROS generated. After APS repair, the fluorescent intensity decreased and this effect in APS1 treatment group was the most obvious. The above results indicated that APSs could reduce ROS production and lower the cellular damage, which were correlated with their Mw.

### Changes of mitochondrial membrane potential after APS repair

**Figure 5:**
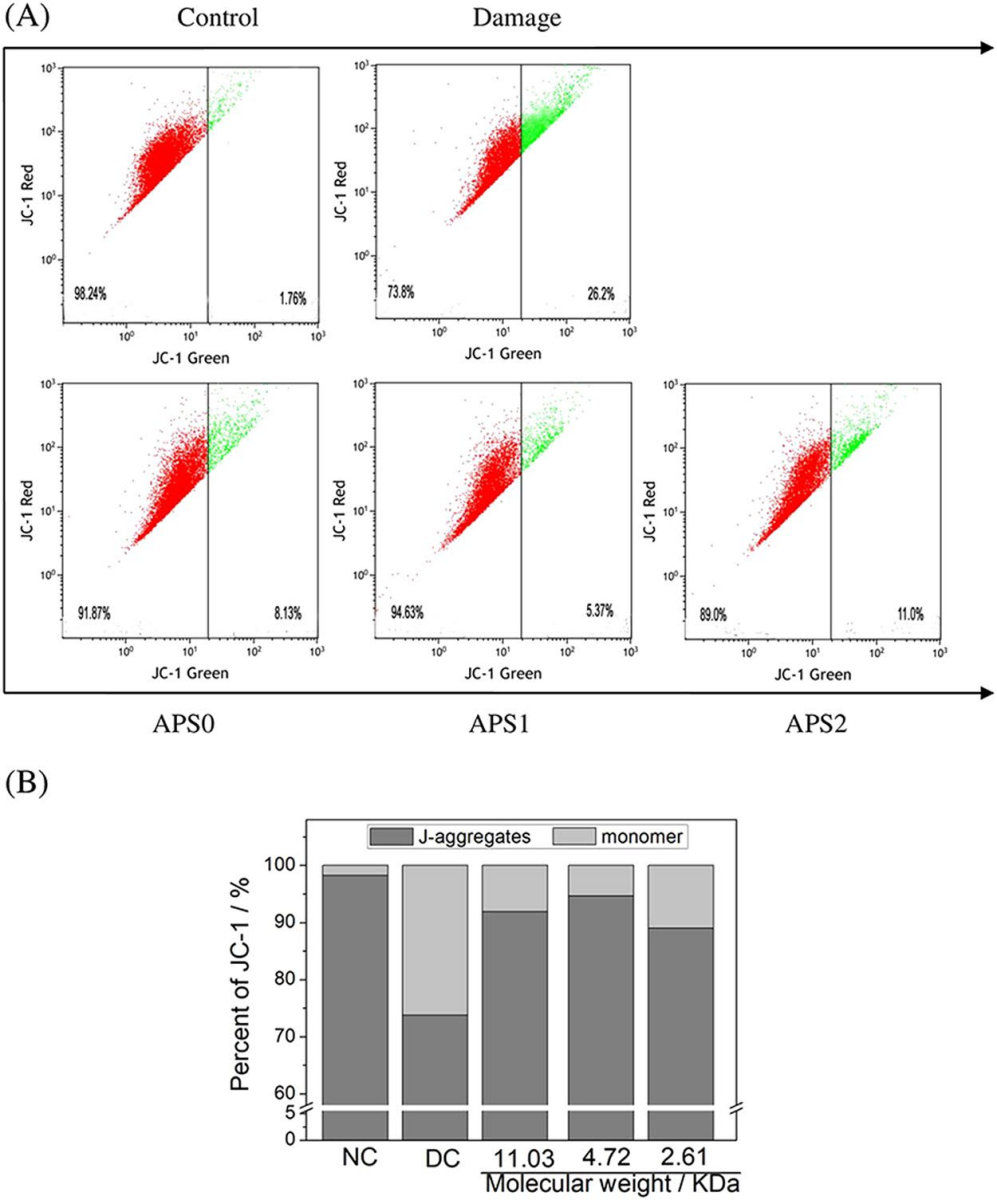
Changes in Δψm of oxalate damaged HK-2 cells after repair by different Mw APS (60 μg/mL) for 10 h. (**A**) Representative dot plot of Δψm; (**B**) the aggregated degree of JC-1. JC-1 monomers (green fluorescence) represented the reduced Δψm.

The loss of ΔΨm is a hallmark of early apoptosis of cells^16^. The changes of Δψm in damaged cells and APS repaired cells were shown in Fig. 5. The percentage of red fluorescence in normal control group was 98.24%. The percentage of red fluorescence reduced to 73.8% when cells were damaged by oxalate, which indicated that Δψm evidently reduced. However, the Δψm in APS0, APS1, and APS2 repaired cells increased in varying degrees. Especially for the APS1-repaired group, the percentage of red fluorescence was 94.67%, which was larger than that in the APS0 (91.87%) and APS2 treatment groups (89.0%). Thus, APS1 presented the best repair ability on the damaged mitochondria among the APSs.

### APS repair improved lysosomal integrity

Acridine orange (AO) is a lysosomal nutrient that easily accumulates in lysosomes through proton capture. AO accumulation changed green fluorescence emission in cytoplasm to red fluorescence emission in lysosome^19,20^.

**Figure 6:**
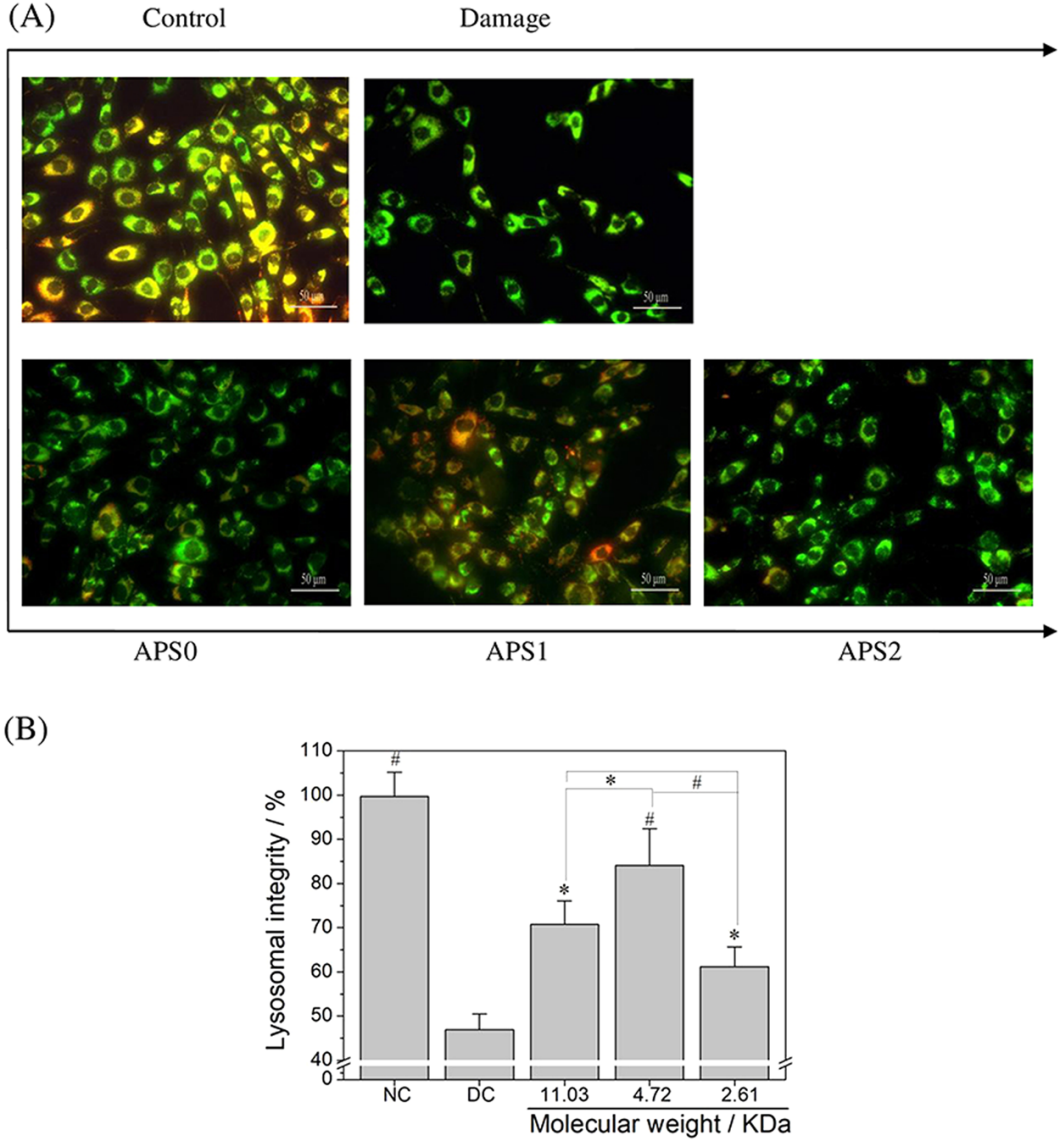
Changes in lysosome integrity of oxalate damaged HK-2 cells after repair by different Mw APS (60 μg/mL) for 10 h. (**A**) The fluorescence microscope images of lysosome integrity. (**B**) Quantitative analysis results of lysosome integrity. Compared with DC group, ^*^P < 0.05, ^#^P < 0.01.

Lysosome structure is complete (100.0% ± 5.5%), and the superposition of red fluorescence and green fluorescence shows strong orange-red in normal cells (Fig. 6). The lysosome integrity in the damaged cells was 46.9% ± 3.6%, which increased to 70.8% ± 5.3%, 81.1% ± 8.3%, and 61.2% ± 4.4% after repair by APS0, APS1, and APS2, respectively. Therefore, APS1 had the greatest repair effect on the lysosomes of cells among the APSs.

### APS repair restored cell cycle progress

**Figure 7:**
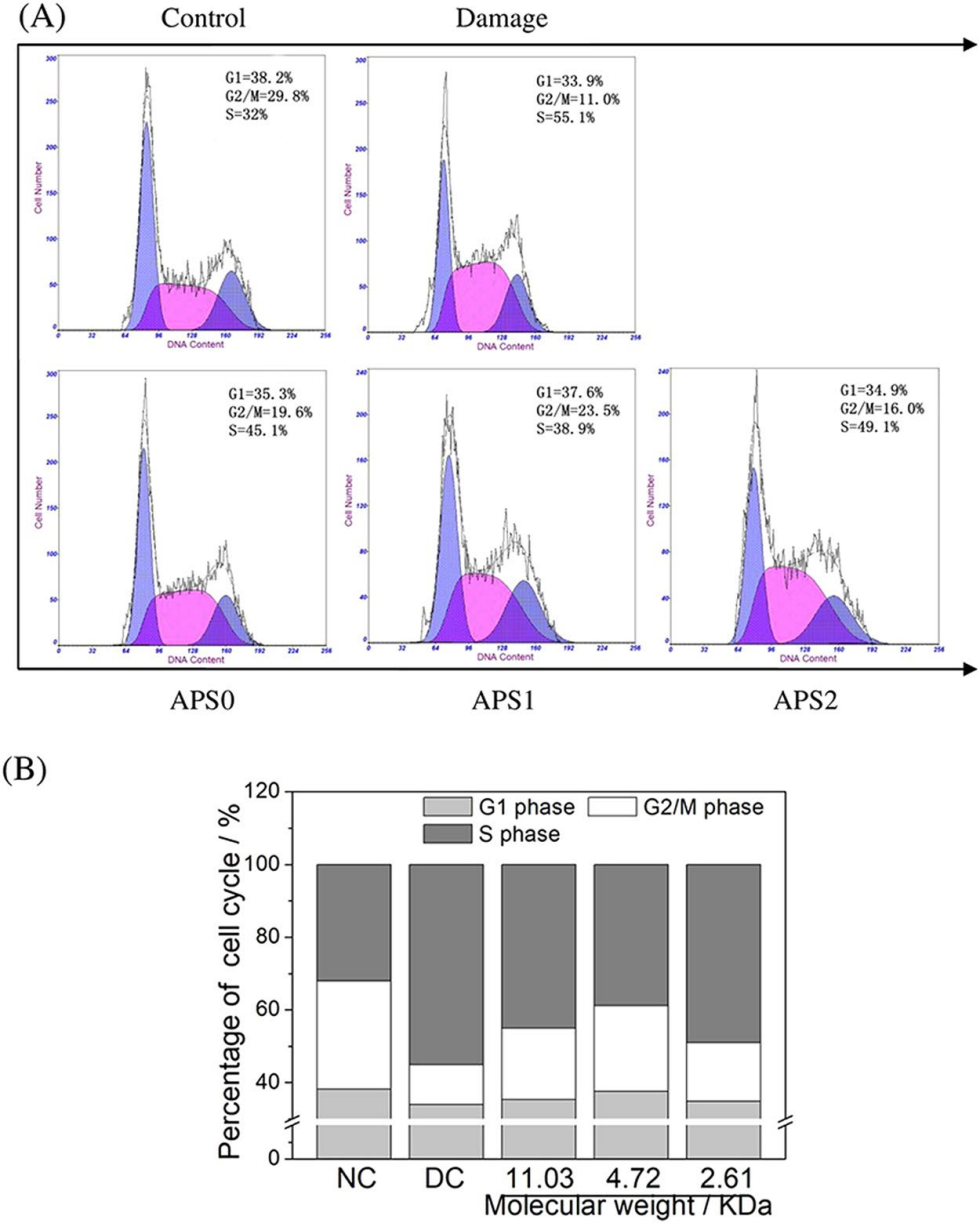
Cell cycle changes of oxalate damaged HK-2 cells after repair by different Mw APS (60 μg/mL) for 10 h. (**A**) Cell cycle results detected by flow cytometry; (**B**) the percentage of cells in the G1, G2/M and S phase.

Propidium iodide (PI) intercalates into double-stranded DNA and fluoresces, and fluorescence intensity is proportional to the amount of double-stranded DNA^21^. When the normal cells were damaged by oxalate, the percentage of S phase cells increased obviously from 32% to 55.1%, and the percentage of G2/M phase cells decreased from 29.8% to 11.0% (Fig. 7). The results showed that cells arrested in the S phase after oxalate damage. After APS0, APS1, and APS2 repair, the number of cells in the S phase declined from 55.1% to 45.1%, 38.9%, and 49.1%, respectively, and the number of cells in the G2/M phase rose from 11.0% to 19.6%, 23.5%, and 16.0%, respectively. Cell cycle arrest reflects the degree of DNA damage^22^. The fewer the cells arrested in S phase, the greater the cell repair effect and the higher the rate of DNA synthesis. Thus, APS1 has the best repair ability on damaged DNA among the APSs (Fig. 7B).

### APS repair decreased cell apoptosis and necrosis

In the early stage of apoptosis, phosphatidylserine (PS) is turned from the inner side of the cell membrane to the surface, and the phospholipid binding protein annexin V can be combined with PS. PI cannot pass through the membrane of normal cells and early-stage apoptotic cells but can penetrate the membrane of apoptotic middle- and late-stage cells and necrotic cells. Thus, a flow cytometric analysis was performed to detect cell apoptosis and necrosis by an annexin V/PI double staining method^23^.

**Figure 8:**
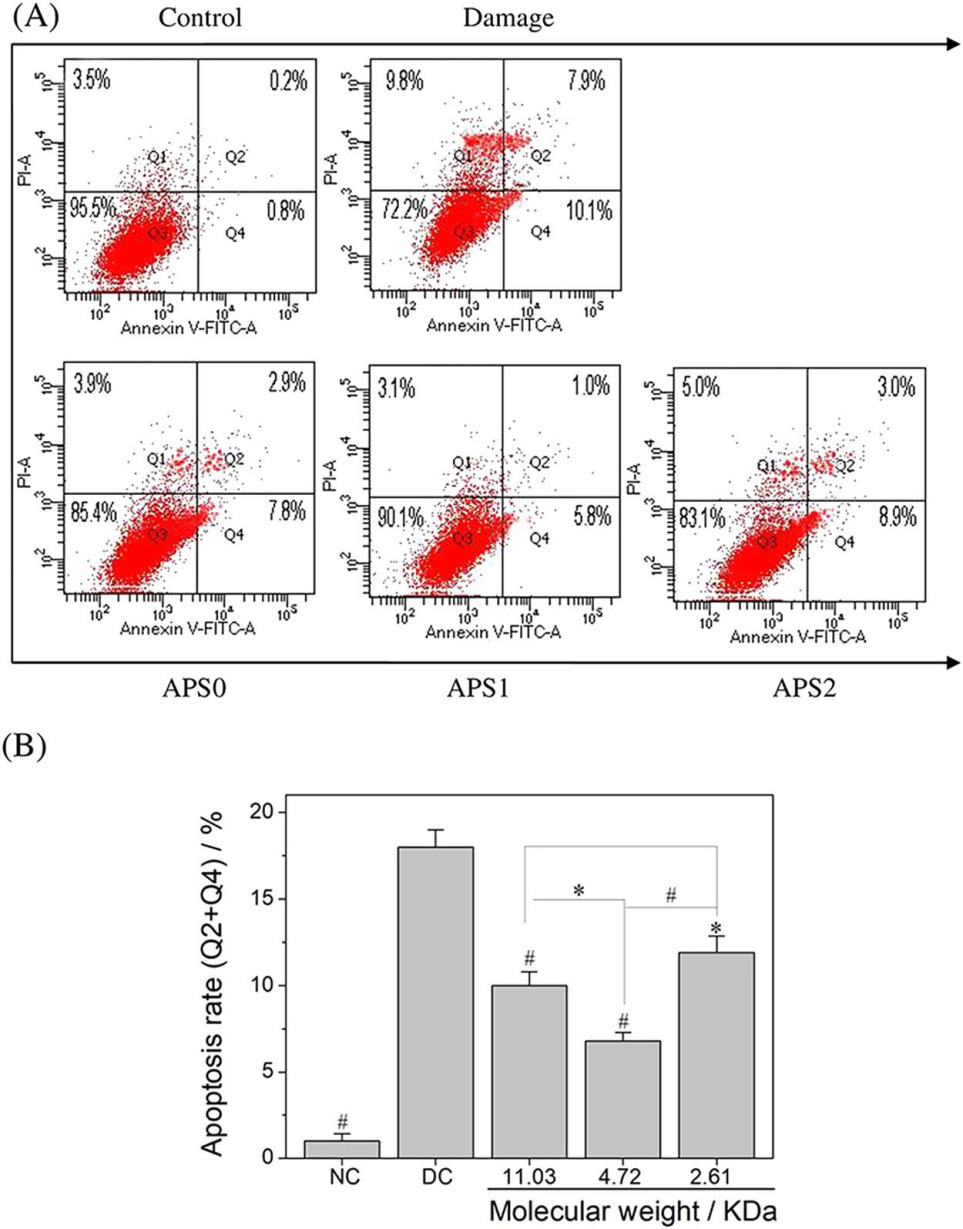
Changes in apoptosis and necrosis rate of oxalate damaged HK-2 cells after repair by different Mw APS (60 μg/mL) for 10 h. (**A**) Cell apoptosis and necrosis rate detected by flow cytometry. (**B**) Quantitative results of apoptosis rate (Q2 + Q4). Compared with DC group, ^*^P < 0.05, ^#^P < 0.01.

Figure 8A showed the dot plot of cell apoptosis and necrosis results. The apoptosis rate (Q2 + Q4) increased from 1% to 18% when the normal cells were injured by oxalate (Fig. 8B). When the damaged cells were repaired by APS0, APS1, and APS2, the apoptosis rates decreased to 10.7%, 6.8%, and 11.9%, respectively, which were lower than 18% of the damaged group. The varying pattern of necrotic rate was similar to that of apoptotic rate. The above results indicated that APSs can reduce cellular apoptosis and necrosis, and APS1 exhibited the best repair effect.

### Effects of APSs on tight junctions in oxalate damaged cells

**Figure 9:**
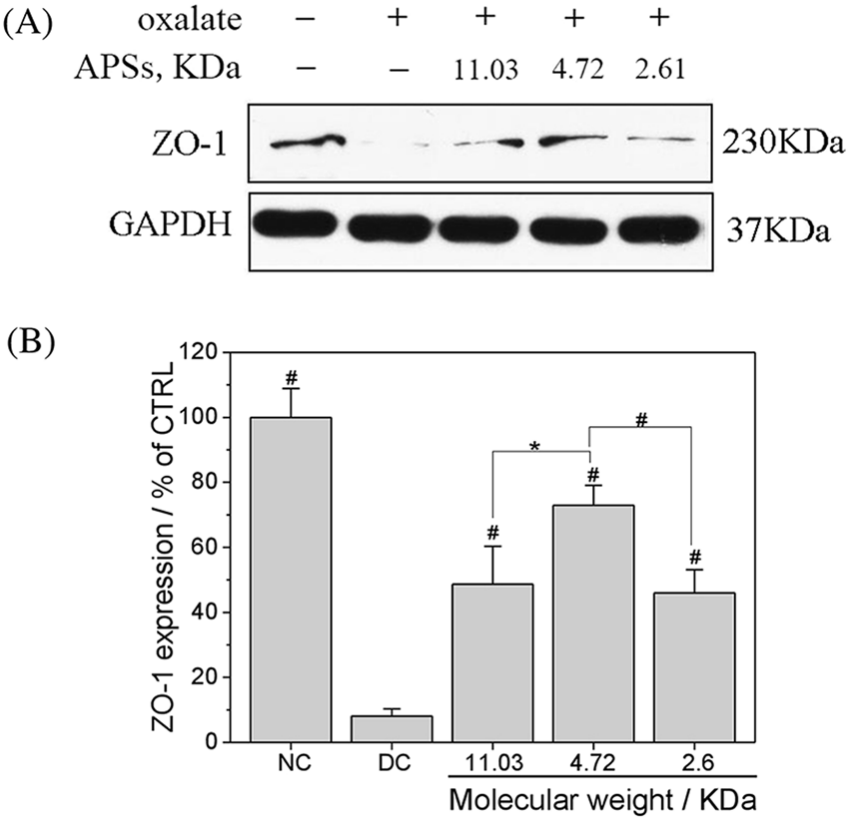
APSs with different Mws influenced the tight junction-associated protein ZO-1. ZO-1 expression was detected by Western blot analysis (**A**) and was quantified by Imagel software (**B**). Compared with DC group, *P < 0.05; ^#^P < 0.01.

To investigate the effects of APS repair on the expression of tight junction protein, we measured the expression of zonula occludens-1 (ZO-1) by Western blot analysis (Fig. 9). Compared with the normal control group, the expression of ZO-1 was significantly down-regulated after oxalate exposure (Fig. 9A). However, the expression of ZO-1 increased obviously after APS0, APS1, and APS2 repair. This result indicated that APSs, especially APS1, can repair the destruction of intercellular junctions caused by oxalate.

### Nrf2-Keap1 pathway

**Figure 10:**
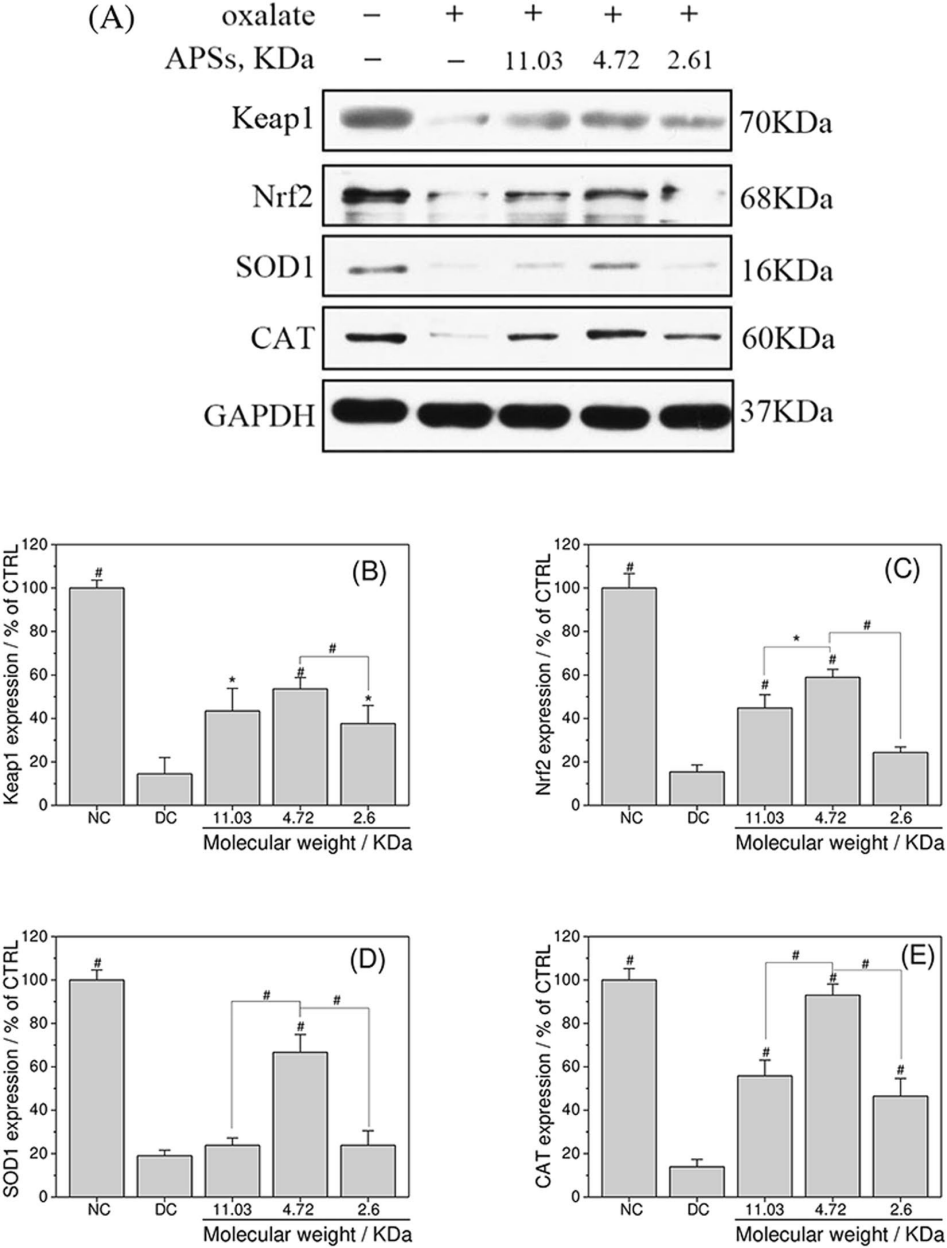
APSs with different Mws influenced the Nrf2–Keap1 signaling pathway. Protein expression of Keap1, Nrf2, SOD1 and CAT were detected by Western blot analysis (**A**). Protein expression of Keap1 (**B**), Nrf2 (**C**), SOD1 (**D**) and CAT (**E**) were quantified by Imagel software. Compared with DC group, *P < 0.05; ^#^P < 0.01.

Activation of the Nrf2–Keap1 signaling pathway is an important way to protect cells from oxidative stress damage. The expression levels of Kelch-like ECH-associated protein 1 (Keap1), nuclear erythroid 2-related factor 2 (Nrf2), superoxide dismutase 1 (SOD1), and catalase (CAT) were detected by Western blot to investigate the repair ability of APSs with various Mws on the oxalate-induced oxidative stress injury of HK-2 cells (Fig. 10A).

The overall expression of Keap1, Nrf2, and downstream antioxidant protein SOD1 and CAT mediated by Nrf2 evidently decreased in the oxalate-induced damage group compared with the normal group (Fig. 10B–E). However, after being repaired by APSs, the expression of Keap1, Nrf2, SOD1, and CAT increased at different degrees but still less than that of the control group. The APS1 with moderate Mw exhibited the best repair effect. Thus, we deduced that APSs inhibit ROS production by activating the Nrf2–Keap1 signaling pathway and ultimately exert their repair function on oxalate-mediated cellular oxidative stress damage.

### Changes of the expression levels of inflammation markers

**Figure 11:**
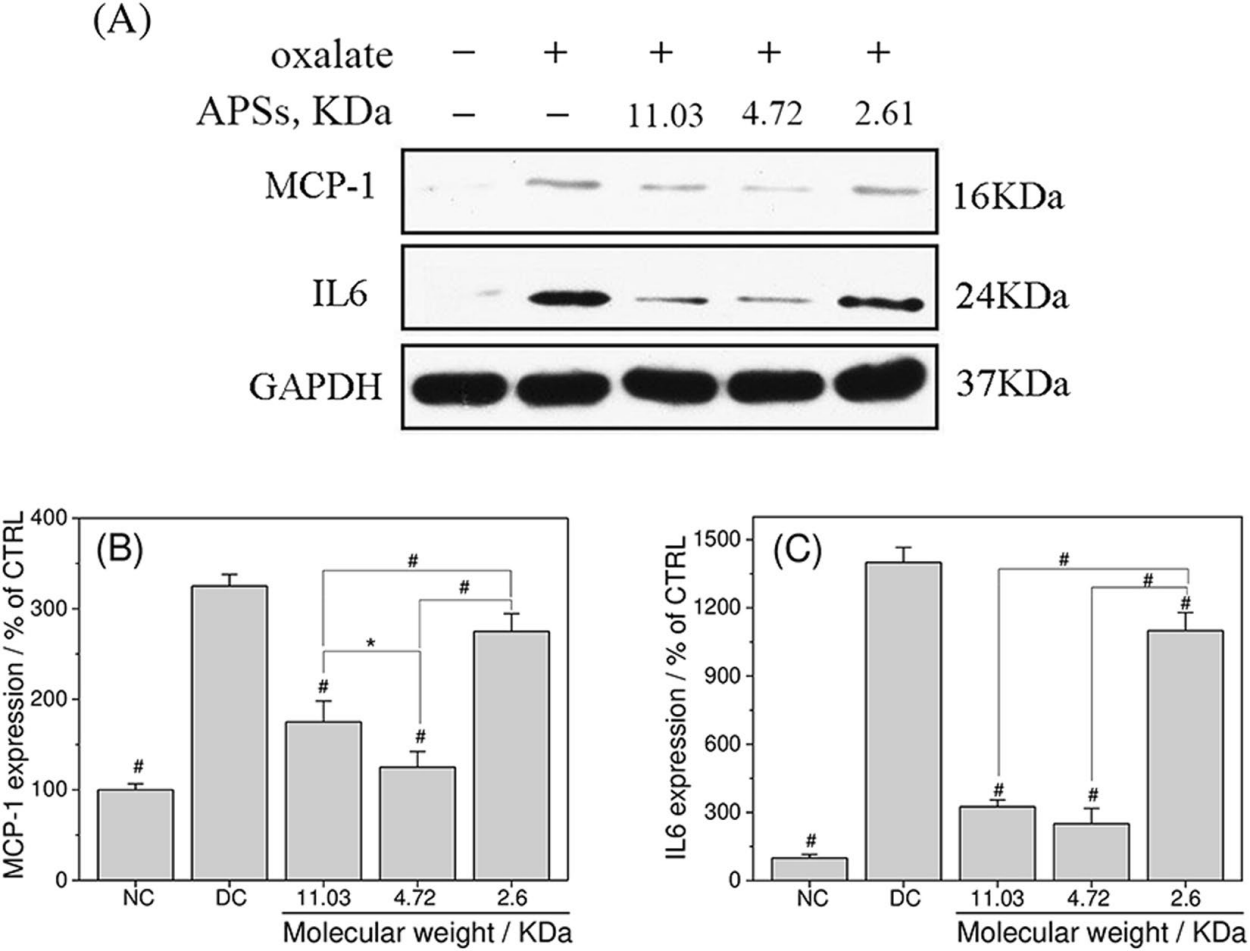
APSs with different Mws inhibited oxalate-induced inflammation level. Protein expression levels of MCP-1 and IL-6 were detected by Western blot analysis (**A**). Protein expression levels of MCP-1 (**B**) and IL-6 (**C**) were quantified by Imagel software.

Damaged renal tubular cells express various cytokines and other mediators of inflammatory response^24^. Therefore, the expression levels of representative inflammation markers, including monocyte chemoattractant protein-1 (MCP-1) and interleukin-6 (IL-6), were investigated. The expression levels of MCP-1 and IL-6 in oxalate-treated cells were obviously higher than those in the control group (Fig. 11A), indicating that oxalate induced inflammation. After being repaired by different Mws of APSs, MCP-1 and IL-6 expression levels decreased gradually. Compared with APS0 and APS2, APS1 exerted the best inflammation inhibition effect (Fig. 11B,C).

## Discussion

### Effects of APS Mw on its biological activity

The Mw of polysaccharides is an important factor that affects their biological activity. The bioactivity for different kinds of polysaccharides with various Mws is different^25^. The above results suggested that APS1 with a Mw of 4.72 KDa possessed stronger repair effect than APS0 (11.03 KDa) and APS2 (2.61 KDa).

High Mw of polysaccharides cannot easily enter the cell membranes to exert their biological effects due to their highly compact molecular structure, large molecular size, and low water solubility^26^. However, active polymeric structure might not be formed in polysaccharide with a very low Mw, causing the decrease of polysaccharide activity^27^, and the hydrogen bond structure of the polysaccharide molecules incurs damage, leading to reduced bioactivity. Only polysaccharide with appropriate Mw possesses high freedom degree and small steric hindrance, which promote their biological activity. Zhang *et al*.^28^ reported that wolfberry (*Lycium barbarum L*.) polysaccharide with a Mw of 10.2 KDa has anticancer ability, whereas wolfberry with a Mw of 6500 KDa does not show anticancer activity. The high-molecular-weight group (413 KDa) of *Pleurotus eryngii* polysaccharide shows better antitumor effect on human hepatoma cells compared with the low-molecular-weight group (12 KDa)^29^.

### Repair mechanism of APSs on oxalate damaged cells

High concentrations of oxalate caused the damage to HK-2 cells and cell integrity loss and reduced the size of cells (Fig. 3). In addition, oxalate exposure caused disruption of cell-cell contact, leading the decrease of the expression of tight junction protein ZO-1. After being repaired by APSs, the cells gradually recovered to normal cell morphology.

After exposure to high concentrations of oxalate, HK-2 cells produced large amounts of ROS (Fig. 4), which can attack cells, affect the normal functions of the cells, induce the damage of cells, and result in cell death^30^. Oxidative damage of cells induced by ROS enhances crystallite adhesion and promotes microlithiasis formation^31^. The ROS level of cells remarkably decreased after APS repair, which indicated that APSs could alleviate the oxidative damage of cells by reducing the production of intracellular ROS.

Mitochondria provide energy for the physiological activities of cells by synthesizing ATP, and ΔΨm is extremely important for mitochondria to maintain their normal function. Thus, the decline of ΔΨm can predict early apoptosis of cells^32^. In the present study, the ΔΨm remarkably increased after repair by APSs (Fig. 5), suggesting that the APSs exerted a good repair effect on damaged mitochondria.

Oxidative stress induced by oxalate affects the stability of lysosomal membranes, which may cause the release of cathepsin from the lysosome into the cytoplasm and thus disrupt lysosomal membrane integrity (Fig. 6)^33^. APSs remarkably improve the integrity of lysosomes and repair damaged lysosomes. The structure of APSs is highly similar to that of glycosaminoglycan (GAG)^34^, which can reduce the production of hydroxyl radicals, inhibit lipid peroxidase, increase the antioxidant capacity of cells, and protect cells from death^35^. The repair mechanism of APSs is similar to that of GAG, which is mainly related to the stabilization of the lysosomal membrane and inhibition of cathepsin release from the lysosome into the cytoplasm^36,37^.

Cell cycle is closely related to cell apoptosis. Factors that cause cell cycle disorders may lead to early cell apoptosis^38^. ROS directly damage DNA molecules, causing single-strand or double-strand breaks and base deletions of DNA, ultimately leading to cell-cycle arrest and altered expression of proteins that inhibit or trigger apoptosis^39^. APSs can inhibit the oxidative damage of DNA by scavenging excess ROS. The number of cells in the S phase remarkably decreased, whereas the number of cells in the G2/M phase increased (Fig. 7) after APS repair. This result indicated that APS accelerated DNA synthesis and promoted cell cycle progression from the S phase to the G2/M phase.

APSs serve their repair function in cells mainly by eliminating excess ROS. The key mechanism of APS-induced anti-oxidant enzyme production is related to the activation of the Nrf2-Keap1 pathway. Nrf2 is an important transcription factor that plays a vital role in protection by maintaining cellular homeostasis through anti-oxidant enzyme expression^40^. Nrf2 is normally maintained in the cytoplasm by interaction with the cytosolic repressor protein Keap1, which promotes the proteosomal degradation of Nrf2^41,42^. Basing from our present results (Fig. 10), we propose that APSs induces Nrf2 by upregulating cellular SOD and CAT and reducing ROS generation. Alternatively, some reactive or electrophilic forms of APS may interact with the cysteine residues present in Keap1, resulting in conformational changes and dissociation of the Keap1–Nrf2 complex^43^. Activation of Nrf2 by APS ultimately increases the levels of antioxidant enzymes through binding between Nrf2 and the antioxidant-response element. Therefore, the activation of the Nrf2 signaling pathway is an important way to protect cells from oxidative stress damage and toxic damage.

Studies have proposed that oxalate stimulates renal epithelial cells to generate chemokines, which consequently attract macrophages and leukocytes that induce localized inflammation^44–46^. The macrophages and giant multinucleated cells release their contents. These events are accompanied by the secretion of the pro-inflammatory cytokine IL-6. Huang *et al*.^24^ used 0.2–2.0 mM oxalate to damage HK-2 cells and found that the expression of IL-6 upregulates with increasing concentration of oxalate. Further results showed that the oxalate-stimulated IL-6 expression and secretion were inhibited by the free radical scavenging agent (SOD), suggesting that oxidative stress is involved in oxalate-induced inflammation.

**Figure 12:**
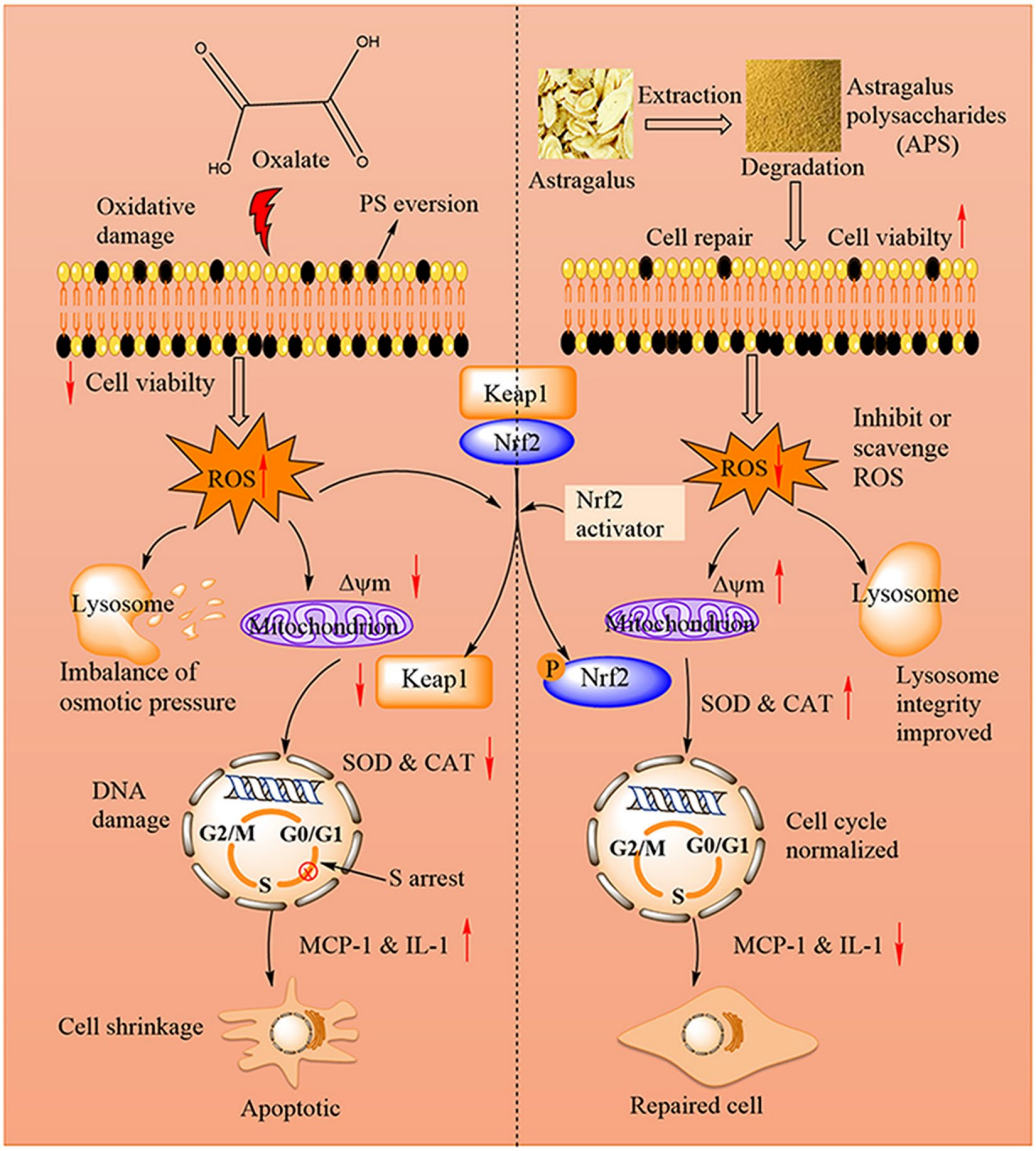
Repair schematic diagram of APSs with different Mws on damaged HK-2 cells.

Basing from the above results, we proposed a repair schematic diagram of APSs with various Mws on damaged HK-2 cells (Fig. 12). After oxalate injury, the viability of normal HK-2 cells decreased, their morphology destroyed, intercellular junctions destructed, active oxygen release increased, lysosomes ruptured, ΔΨm decreased, cell cycle arrested, and inflammatory factor (MCP-1 and IL-6) released. APS repair can enhance ΔΨm, repair lysosomal integrity, promote DNA synthesis, and accelerate the transformation of the S phase to the G2/M phase by eliminating excessive ROS production in cells via the activation of Nrf2-Keap1 pathway, leading to anti-oxidant enzyme (SOD1 and CAT) production, recovering cell morphology and tight junctions, thereby inhibiting cell death and inflammatory. During cell apoptosis, PS can be flipped to the surface of the cell membrane, which provides active sites for the nucleation and growth of CaOx crystals on the cell surface. Hence, APSs, especially APS1 with a moderate Mw, exert good drug activity in the prevention or treatment of renal stones.

## Conclusion

All three APSs (APS0, APS1, and APS2) with respective Mws of 11.03, 4.72, and 2.61 KDa presented repair effect on oxidatively damaged HK-2 cells. For the APS repaired groups, cell morphology recovered, cell viability strengthened, ROS production reduced, ΔΨm and lysosome integrity improved, cell-cycle progression gradually normalized, and apoptosis rate decreased compared with the damaged group. All three APS could promote the expression of Keap1, Nrf2, SOD1 and CAT, and inhibit MCP-1 and IL-6 expression. APSs exert their repair function through activation of the Nrf2-Keap1 signaling pathway and inhibition of inflammation. The repair effect of APSs was in the order of APS1 > APS2 > APS0, which indicated that APS1 with a moderate Mw presented the optimal repair on damaged HK-2 cells. Our results suggested that degraded APS fractions, especially APS1, have great potential to act as candidate drugs for the prevention or treatment of kidney stone formation.

## Supplementary information

Supplementary Information

## Data Availability

All data generated or analyzed during this study are included in this manuscript (and its Supplementary Information file).
