# Supplementary material for: Repair Effects of Astragalus Polysaccharides with Different Molecular Weights on Oxidatively Damaged HK-2 Cells
Source: Sci Rep. 2019 Jul 8;9:9871. doi: 10.1038/s41598-019-46264-y (PMC6614371; doi:10.1038/s41598-019-46264-y)
Supplement: Supplementary file 1 — Supplementary Information [file 41598_2019_46264_MOESM1_ESM.pdf]

Supplementary Information

Repair Effects of Astragalus Polysaccharides with Different Molecular Weights on Oxidatively Damaged HK-2 Cells

Jin Han[1], Da Guo[2], Xin-Yuan Sun[2], Jian-Min Wang[2], Jian-Ming Ouyang[2]*, Bao-Song Gui[1]*

Affiliations

[1] Department of Nephrology, the Second Hospital of Xi'an Jiaotong University, Xi'an 710004, China

[2] Institute of Biomineralization and Lithiasis Research, Jinan University, Guangzhou 510632, China

Full-length immunoblots from Figure 9A

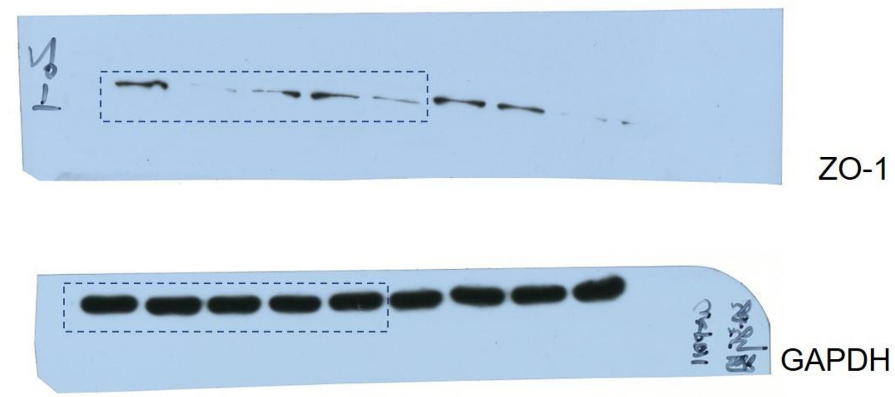

Figure S9A. APSs with different Mws influenced the tight junction-associated protein ZO-1. ZO-1 expression was detected by Western blot analysis (A).

Full-length immunoblots from Figure 10A

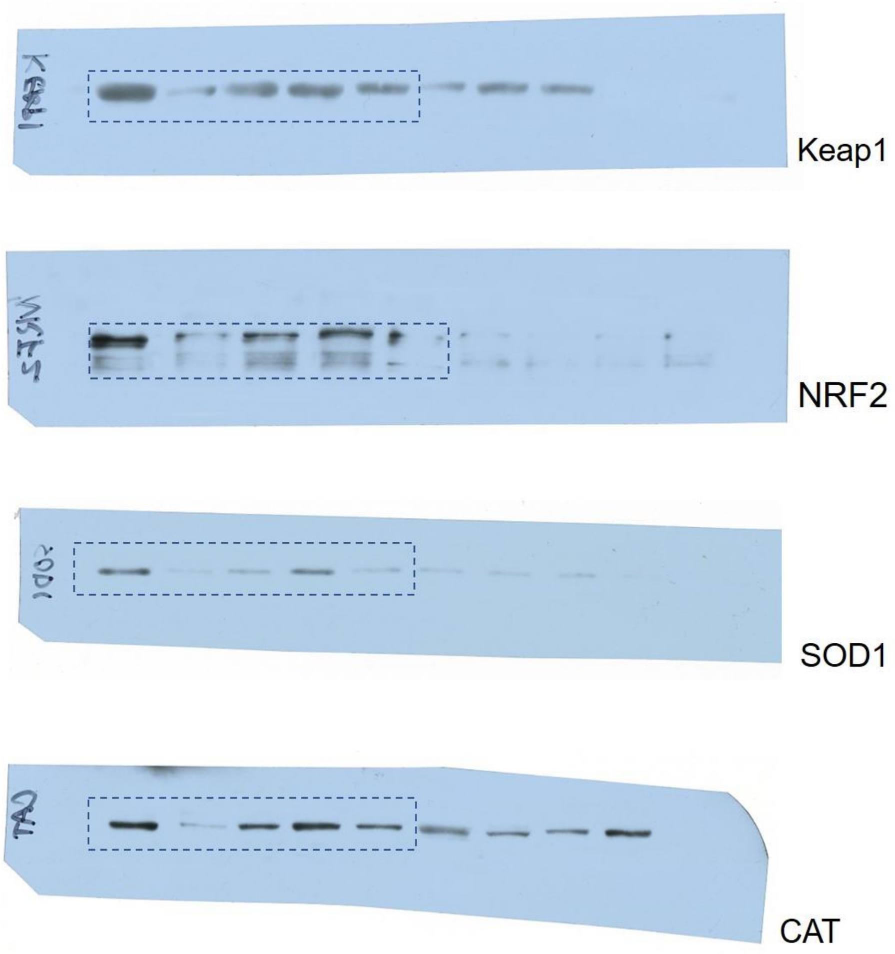

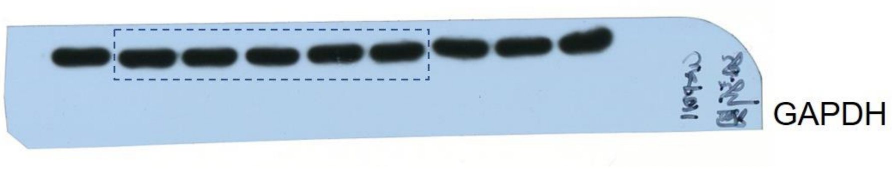

Figure S10A. APSs with different Mws influenced the Nrf2–Keap1 signaling pathway. Protein expression of Keap1, Nrf2, SOD1 and CAT were detected by Western blot analysis (A).

Full-length immunoblots from Figure 11A

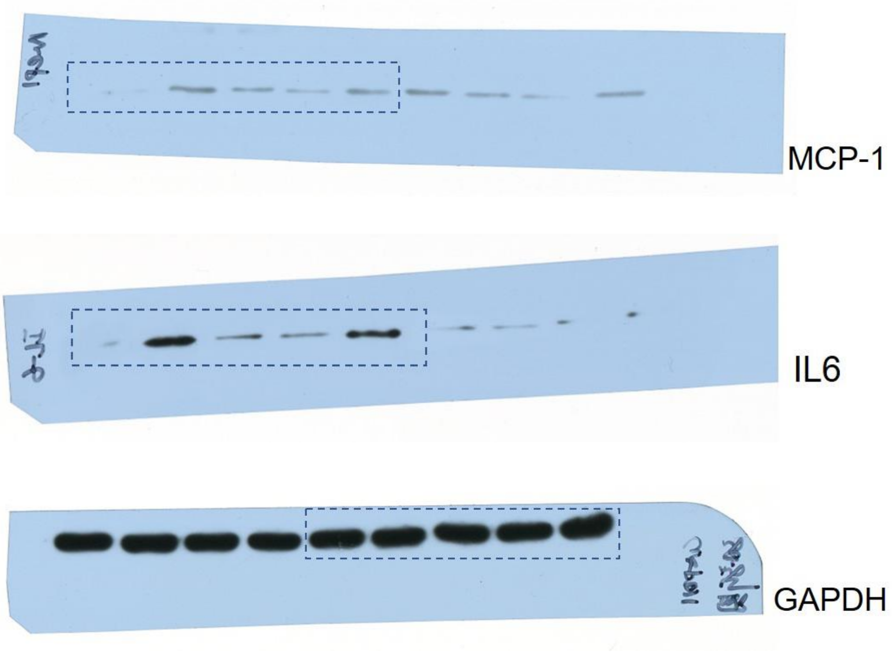

Figure S11A. APSs with different Mws inhibited oxalate-induced inflammation level. Protein expression levels of MCP-1 and IL-6 were detected by Western blot analysis (A).
